# Pre-hospital critical care management of severe hypoxemia in victims of Covid-19: a case series

**DOI:** 10.1186/s13049-021-00831-3

**Published:** 2021-01-12

**Authors:** Jens Otto Mæhlen, Roger Mikalsen, Hans Julius Heimdal, Marius Rehn, Jostein S. Hagemo, William Ottestad

**Affiliations:** 1 Air Ambulance Department, Oslo University Hospital, Oslo, Norway; 2 Department of Anesthesiology, Akershus University Hospital, Sykehusveien 25, 1478 Lørenskog, Norway; 3 Air Ambulance Department, Vestre Viken, Ål, Norway; 4 Department of Research and development, Norwegian Air Ambulance Foundation, Oslo, Norway; 5 Faculty of Health Sciences, University of Stavanger, Stavanger, Norway; 6 Faculty of Medicine, University of Oslo, Oslo, Norway

**Keywords:** Prehospital, Covid-19, Hypoxemia, Case series, Non-invasive ventilation

## Abstract

**Objective:**

Despite critical hypoxemia, Covid-19 patients may present without proportional signs of respiratory distress. We report three patients with critical respiratory failure due to Covid-19, in which all presented with severe hypoxemia refractory to supplemental oxygen therapy. We discuss possible strategies for ventilatory support in the emergency pre-hospital setting, and point out some pitfalls regarding the management of these patients. Guidelines for pre-hospital care of critically ill Covid-19 patients cannot be established based on the current evidence base, and we have to apply our understanding of respiratory physiology and mechanics in order to optimize respiratory support.

**Methods:**

Three cases with similar clinical presentation were identified within the Norwegian national helicopter emergency medical service (HEMS) system. The HEMS units are manned by a consultant anaesthesiologist.

Patient’s next of kin and the Regional committee for medical and health research ethics approved the publication of this report.

**Conclusion:**

Patients with Covid-19 and severe hypoxemia may pose a considerable challenge for the pre-hospital emergency medical services. Intubation may be associated with a high risk of complications in these patients and should be carried out with diligence when considered necessary. The following interventions are worth considering in Covid-19 patients with refractory hypoxemia before proceeding to intubation. First, administering oxygen via a tight fitting BVM with an oxygen flow rate that exceeds the patient’s ventilatory minute volume. Second, applying continuous positive airway pressure, while simultaneously maintaining a high FiO_2_. Finally, assuming the patient is cooperative, repositioning to prone position.

## Introduction

Covid-19 is characterized by an initial phase of unspecific symptoms such as fever and unproductive cough [1]. Due to risk of disease transmission, patients are advised to stay quarantined at home, unless medical care is needed. However, some patients develop severe respiratory failure and extreme hypoxemia, yet without proportional signs of respiratory distress or air hunger. Although anecdotal, a common clinical pattern has emerged; a remarkable discrepancy between relatively well-preserved lung compliance and severely compromised pulmonary gas exchange [2, 3]. A brisk ventilatory response with a rapid respiratory rate has been reported to lead to hypocapnia in some of these patients [4], possibly contributing to lack of air hunger [5]. Apart from a rapid respiratory rate, the clinical presentation in these patients may be misleading. Despite critical hypoxemia these patients tend to be awake, responsive, cooperative and hemodynamically stable. This particular clinical presentation has been coined “silent hypoxemia” [3]. In this pre-hospital case-series we report three Covid-19 patients with critical respiratory failure and discuss possible strategies for ventilatory support.

## Methods

The Norwegian national air ambulance system is governmentally funded and consist of 12 Helicopter Emergency Medical Services (HEMS) bases. These air ambulance units are all manned by a consultant anesthesiologist and a rescue man. Relevant cases were identified from clinical governance discussions and the attending consultant provided a focused narrative of the events. Publication of these case reports has been approved by the Regional committee for medical and health research ethics (Ref. ID: 142091) and written informed consent has been obtained from next of kin. The exact age of the patients remains undisclosed for privacy protection purposes.

### Pre-hospital Covid-19 cases

#### Case 1

A man in his sixties presented to his general practitioner with tachypnea, high fever and a dry cough. The patient was admitted to hospital and ambulance transport was initiated. Despite receiving supplemental oxygen, the patient’s pulse oximetry readings decreased from 72 to 55% and the paramedics requested HEMS assistance. Upon HEMS arrival, the patient was awake and cooperative. The patient had been placed in an upright position. He was administered intravenous paracetamol due to a body temperature of 40 °C. As the patient refused wearing a non-rebreather mask he received oxygen via a nasal cannula at a flow rate of 10 l/minute. Physical examination revealed a respiratory rate of 20–25 breaths/minute, a peripheral oxygen saturation (SpO_2_) of 55%, a heart rate of 80 beats/minute, a blood pressure of 120/60 mmHg, slightly cold peripheries and a body temperature of 38.9 °C. Although tachypneic, he was able to speak in whole sentences. As the patient was alert and cooperative, no further treatment was initiated. During transport the patient accepted that the nasal cannula was replaced with a non-rebreather with a flow rate of 12 l/minute. However, his SpO_2_ did not improve. His condition remained unchanged upon hospital arrival 1 h later. He was admitted to the intensive care unit (ICU) where treatment with non-invasive ventilation (NIV) was initiated. After a few hours of NIV treatment the patient was intubated and mechanically ventilated due to exhaustion. He was later diagnosed with Covid-19 and died in the ICU approximately 3 weeks later.

#### Case 2

A woman in her fifties with a previous history of hypertension, asthma and obesity presented with fever and a dry cough. One of her family members had tested positive for SARS-CoV-2. Her condition had deteriorated rapidly the last hour with worsening dyspnea. The paramedic crew arriving on scene requested HEMS assistance as the patient remained severely hypoxemic after receiving supplemental oxygen. Upon HEMS arrival, the patient presented with severe cyanosis and labored breathing. Despite this she was awake and cooperative. Physical examination revealed a respiratory rate of 50 breaths/minute, SpO_2_ of 52% while breathing 12 l/minute oxygen on a non-rebreather mask, heart rate of 142 beats/minute, blood pressure 200/110 mmHg, cold peripheries and a body temperature of 39 °C. On route to hospital the non-rebreather mask was replaced with a bag valve mask (BVM) which was sealed tight around the patients mouth and nose. The oxygen flow rate administered was unchanged at 12 l/minute. A positive end-expiratory pressure (PEEP) valve was connected to the BVM. The SpO_2_ increased from 52 to 66% and the respiratory rate remained unchanged with an end-tidal CO_2_ of 4.2 Kilopascal (kPa). As she became increasingly exhausted, she received assisted ventilation with the BVM. Shortly after arrival to the hospital she was intubated and the patient suffered temporary cardiac arrest during the procedure. She was later diagnosed with Covid-19 and died in the ICU a few weeks later.

#### Case 3

A man in his fifties with a past history of hypertension, obesity and cerebrovascular disease was in home-quarantine due to symptoms of Covid-19. An ambulance was requested as his condition quickly deteriorated with increasing respiratory distress. Upon arrival the paramedics found the patient severely hypoxemic. They initiated treatment with a disposable CPAP system (pulmodyne O_2_-Max®), but was terminated due to lack of improvement and replaced by a non-rebreather mask with a flow rate of 10 l/minute. A HEMS unit was requested for assistance, and the physician found the patient awake and cooperative, but with severe dyspnea. Physical examination revealed a respiratory rate of 50 breaths/minute, SpO_2_ 35% while breathing oxygen 10 l/minute on a non-rebreather mask and a blood pressure of 120/80 mmHg. The patient was placed in a semi-upright position, the non-rebreather mask was removed and a BVM with PEEP of 10 cmH_2_O and oxygen flow rate of 15 l/minute was applied. SpO_2_ increased from 35 to 50%. The decision to perform a prehospital intubation was made since the patient responded poorly to oxygen therapy and due to exhaustion could not longer sustain adequate minute ventilation.

Prior to induction of anesthesia the patient became unresponsive, and assisted ventilations were given. Induction of anesthesia was performed with 250 micrograms fentanyl, 250 mg ketamine and 100 mg rocuronium. The patient was ventilated in the apneic phase and intubated using direct laryngoscopy. First pass intubation was achieved quickly without further drop in SpO_2_. During loading into the ambulance, the patient became pulseless and CPR was commenced. A carotid pulse was identified after approximately 30 s of CPR. A high minute ventilation with the BVM was maintained so that end-tidal CO_2_ remained unchanged at 2.5 kPa. The oxygen saturation increased from 50 to 60%. Systolic blood pressure was 90 mmHg. The patient was positioned in a semi-upright position in the ambulance during transport. Shortly after hospital arrival the patient suffered circulatory arrest with pulseless electrical activity. Despite 25 min of CPR a refractory asystole developed. The patient was diagnosed with Covid-19 post mortem.

## Discussion

We describe three Covid-19 cases presenting with critical respiratory failure managed in the pre-hospital setting. The cause of hypoxemia in these patients is a matter of speculation, and several mechanisms have been suggested. Two recent autopsy studies have reported interstitial thickening and congested capillaries, making both diffusion failure and a low ventilation/perfusion (V/Q) ratio secondary to dead space ventilation plausible mechanisms [6, 7]. However, CT imaging studies also report consolidations in the majority of patients with Covid-19 pneumonia [8]. Consolidations may accordingly contribute to a substantial shunt fraction, which explains the failure of oxygen therapy in some of these patients. Prone positioning has been reported to improve oxygenation in COVID-19 patients [9], lending support to the notion that pulmonary shunting is a contributing factor to hypoxemia. Furthermore, Gatinoni et al. postulated dysregulation of pulmonary vascular tone with loss of hypoxic vasoconstriction and potentially increased pulmonary shunting [10]. Covid-19 patients are reported to have near normal lung compliance and consequently they can maintain large minute volumes for a prolonged period of time without succumbing to exhaustion.

Despite being severely hypoxemic all three patients described in this case series were responsive and cooperative upon presentation. All cases presented with low pulse oximetry values in the 35–60% range. However, how well this reflects arterial oxygen saturation (SaO_2_) is uncertain since pulse oximetry is not validated for SaO_2_ values below 70% and tends to overestimate hypoxemia below this threshold [11, 12].

The patient described in case three suffered transient cardiovascular collapse immediately following intubation. There are many possible explanations why intubating these patients is considered high risk. Initially, several guidelines advised against mask ventilating these patients through the apnoeic period prior to laryngoscopy due to the risk of aerosolization. This may however lead to an unnecessary long apnea time potentially causing a critical drop in oxygen saturation. In line with this, some international guidelines now recommend mask ventilation through the apneic period [13]. Another possible mechanism by which apnea leads to a rapid deterioration in oxygen saturation is the effect of hypercapnia. In Covid-19 patients with a high minute ventilation, hypocapnic hypoxia causes a left shift in the oxygen dissociation curve due to an increase in haemoglobin oxygen affinity and consequently a higher SaO2 for a given PaO2 compared to the normocapnic state. In the case of apnea during induction of anaesthesia, PaCO_2_ will increase, leading to decreased haemoglobin oxygen affinity at the level of the lung and consequently a sudden drop in SaO_2_ [5]. A simultaneous drop in coronary perfusion pressure secondary to the vasodilatory and cardiodepressive effects of the anesthetic drugs, may contribute to hemodynamic collapse.

Early intubation of Covid-19 patients have been recommended by some institutions, however this strategy has recently been challenged [14]. Anecdotal evidence suggest that favorable outcome can be achieved with NIV support even in patients with severe respiratory failure and persistent hypoxemia [15], and that NIV may reduce the need for intubation [16]. Several factors can contribute to a favorable outcome with NIV. First, as these patients seem to have near normal lung compliance, they do not necessarily have increased work of breathing implying that they can endure long periods with NIV support, without the need for heavy sedation. Second, prone positioning has been reported to be effective in Covid-19 and this can be easier to accomplish in awake patients on NIV compared to intubated patients which demands heavy sedation [9]. Further, profound hypoxemia, hypotension and even cardiac arrest have been reported in a significant number of Covid-19 patients during induction of anesthesia and tracheal intubation [17]. We argue that the decision to intubate Covid-19 patients in the pre-hospital setting should rely on a careful risk-benefit analysis, and one should consider NIV in patients with severe hypoxemia. World Health Organization guidelines now suggest a trial of NIV before proceeding to invasive mechanical ventilation in selected patients [18].

Oxygen therapy either as continuous flow or by CPAP failed to relieve our patients from severe hypoxemia, and we speculate that limitations to oxygen flow-rate and mask seal contributed to the failure of oxygen therapy. Non-rebreather masks, disposable CPAP systems and BVMs will dilute oxygen with a highly variable volume of ambient air, depending on the patient’s minute ventilation, oxygen flow rate and degree of mask seal. In a study in healthy volunteers the FiO_2_ obtained with a non-rebreather mask with oxygen flow of 15 l/minute was approximately 0.6 and fell rapidly with increasing respiratory rate and tidal volume [19]. In the pre-hospital context many emergency services rely on simple disposable CPAP systems powered by oxygen flow through a simple venturi valve. Unfortunately, disposable CPAP systems generally fail to deliver a FiO_2_ ≥ 0.55 [20] and we speculate that this contributed to the ineffective use of CPAP in one of our patients. Adding extra oxygen via a nasal cannula underneath the CPAP may improve the FiO_2,_ however this set up requires two oxygen sources. A mechanical transport ventilator in CPAP mode could permit a FiO_2_ close to 1.0 as these ventilators draw oxygen to match the patients inspiratory flow rate without the need for ambient air. Additionally, a mechanical ventilator may sustain a more stable positive airway pressure. The application of ventilator obtained CPAP has shown to be effective in improving hypoxemia in Covid-19 patients [15], however the time required from onset of CPAP-treatment to an increase in oxygen saturation has not been reported. As an alternative to a CPAP system, a PEEP valve may be connected to the BVM. However, we would like to stress that a BVM with a PEEP valve is not able to generate a continuous positive airway pressure, but only applies positive airway pressure during expiration. The FiO_2_ delivered via a BVM is variable depending on the manufacturer. One study reported a FiO_2_ in the range of 0.43–0.54 at flow rates of 10–15 l/minute, but at higher oxygen flow rates a FiO_2_ ≥ 0.9 was achieved [21]. We would like to highlight two factors which must be taken into consideration in order to deliver a high FiO_2_ with the BVM. First, a tight seal between the patients face and the mask is necessary in order to avoid ambient air being drawn in during inspiration. Second, the oxygen flow rate must exceed the patient’s minute ventilation. If not, the reservoir bag collapses, and ambient air will be pulled into the bag diluting the oxygen and reducing the FiO_2_. The Covid-19 patients reported in this paper responded poorly to oxygen therapy, and due to their high minute ventilation we speculate that the oxygen flow rates delivered were not sufficient to avoid dilution with ambient air.

Hypoxemic patients may fail to respond adequately to supplemental oxygen despite a high FiO_2,_ reflecting a considerable shunt fraction or compromised diffusion. Reducing the shunt fraction can be achieved by recruiting non-ventilated lung volume, as already discussed by applying a positive airway pressure or by facilitating redistribution of perfusion to areas of the lung with better ventilation. Several non-invasive interventions can be applied in order to reduce shunt fraction, and we will discuss prone positioning and inhaled nitric oxide.

Prone position has been reported to improve outcome in intubated patients with acute respiratory distress syndrome [22]. Covid-19 patients have been reported to have dorsal consolidations whereas the ventral parts of the lungs are less affected [8, 23], therefore proning contributes to redistribution of blood from atelectatic areas of the lungs to less atelectatic ventral lung tissue. Proning may also help in recruitment of dorsal atelectasis and mobilization of secretions and thereby preventing further atelectasis. Caputo et al., reported the effects of awake prone position in 50 Covid-19 patients presenting to the emergency department and a majority showed improvement in oxygenation within 5 minutes [9]. Awake prone positioning for hypoxemic Covid-19 patients has already been implemented in some EMS protocols in the United States [24].

Administering inhaled nitric oxide can be logistically challenging in a pre-hospital setting. In situations where the appropriate equipment is readily available, this could be a valuable adjuvant in patients with refractory hypoxemia. A pilot study from 2004 reported that inhaled nitric oxide had an immediate effect on oxygenation in spontaneously breathing SARS patients with hypoxemic respiratory failure [25]. A randomized controlled trial investigating the effects of inhaled nitric oxide in mechanically ventilated patients with Covid-19 is underway [26].

## Conclusion

Patients with Covid-19 and severe hypoxemia may pose a considerable challenge for the pre-hospital emergency medical services. We have reported three Covid-19 patients with severe hypoxemia that proved refractory to oxygen therapy delivered by continuous flow or a disposable CPAP system. Hypoxemic Covid-19 patients with a high minute ventilation demand high oxygen flow rates in order to avoid dilution of oxygen with ambient air, which is difficult to achieve with non-rebreathers and disposable CPAP systems. Intubation may be associated with a high risk of complications in these patients and should be carried out with diligence when considered necessary. The following interventions are worth considering in Covid-19 patients with refractory hypoxemia before proceeding to intubation. First, administering oxygen via a tight fitting BVM with an oxygen flow rate that exceeds the patient’s ventilatory minute volume. Second, applying continuous positive airway pressure, while simultaneously maintaining a high FiO_2_. Finally, assuming the patient is cooperative, repositioning to prone position.

## Data Availability

Not applicable.
